# Role of illness severity scores in predicting mortality in the coronary care unit

**DOI:** 10.1186/cc11021

**Published:** 2012-03-20

**Authors:** G Argyriou, J Terrovitis, G Sainis, V Papas, C Marvaki, S Nanas, C Routsi

**Affiliations:** 1 Medical School, University of Athens, Greece

## Introduction

Several illness severity scores have been developed in order to predict outcome in multidisciplinary ICUs. However, the role of these scores has not been thoroughly investigated in coronary care units (CCUs) and the results are conflicting [1,2]. The aim of this study was to evaluate the utility of two of the most widely used scores - that is, APACHE II and Sequential Organ Failure Assessment (SOFA) - for the prediction of mortality in patients admitted to CCUs.

## Methods

All patients consecutively admitted to an eight-bed CCU from April 2010 to May 2011 were prospectively studied. Demographic, clinical and laboratory data were recorded. Illness severity on admission was measured using the APACHE II and SOFA scores. For the calculation of the scores, the worst values for each variable on admission day were used.

## Results

A total of 200 patients (age 70 ± 17 years, 65% males) were admitted to the CCU during the study period; diagnoses included acute coronary syndrome (65%), pulmonary edema (11.5%), congestive heart failure (5.5%) and other (18%). The median length of CCU stay was 5 ± 3 days and the median length of hospital stay 9 ± 7 days. The CCU mortality rate was 20% and in-hospital mortality 24.2%. Both APACHE II and SOFA scores were independently associated with mortality (OR = 1.30; CI: 1.21 to 1.40, *P *< 0.001 and OR = 1.82; CI: 1.53 to 2.16, *P *< 0.001 respectively). The receiver operating characteristic curves confirmed both scores as equally effective predictors of clinical outcome with areas under the curve of 0.92, *P *< 0.001 and 0.91, *P *< 0.001 for APACHE II and SOFA score respectively.

## Conclusion

The APACHE II and SOFA scores on admission are independent predictors of mortality in patients hospitalized in a CCU. Both scores demonstrate excellent performance in discriminating high-risk patients and thus are useful tools to predict clinical outcome in CCUs.
